# Towards successful implementation of public health research into practice: Experiences and lessons learned from EDUCATE

**DOI:** 10.1016/j.puhip.2023.100447

**Published:** 2023-10-27

**Authors:** Harriet Fisher, Suzanne Audrey, Tracey Chantler, Matthew Dominey, Karen Evans, Lizzie Henden, Matthew Hickman, Louise Letley, Alix Towson, Clare Thomas

**Affiliations:** a National Institute for Health Research Health Protection Research Unit (NIHR HPRU) in Behavioural Science and Evaluation (BSE), Bristol Medical School, University of Bristol, Bristol, UK; b National Institute for Health Research Health Protection Research Unit (NIHR HPRU) in Vaccinations and Immunisation, London School of Hygiene and Tropical Medicine, Keppel Street, London, UK; c NHS England South West, Taunton, UK; d School-aged Immunisation Team, Sirona Care & Health, Bristol, UK; e Communities and Public Health Team, Bristol City Council, Bristol, UK; f Immunisation and Vaccine Preventable Diseases Division, UK Health Security Agency, London, UK; g The National Institute for Health Research Applied Research Collaboration West (NIHR ARC West) at University Hospitals Bristol and Weston NHS Foundation Trust, Bristol, UK

**Keywords:** HPV vaccine, Public health practice, Education, Young people, Implementation, Knowledge mobilisation

## Abstract

**Background:**

The English schools-based human papillomavirus (HPV) vaccination programme is routinely offered to all young people aged 12–13 years. The EDUCATE lesson was developed to overcome barriers to uptake related to unmet information needs by providing young people with information and answering questions they may have about the HPV vaccine. The resource comprises a PowerPoint presentation, interspersed with five short films and a guidance document for professionals delivering the lesson. Adopting public health research into practice is challenging and few papers describe the process. This paper reports the initial use of the EDUCATE resource in schools and the process involved in supporting wider implementation.

**Study design:**

Implementation and knowledge mobilisation.

**Methods:**

Five secondary schools supported implementation of the EDUCATE resource. Delivery took place during April and December 2022 and was observed in four schools, with feedback obtained from two school staff members and 15 young people. Alongside this, meetings were held with over 80 stakeholders with the aim of identifying possible policy levers to encourage use of the EDUCATE resource, and to enhance understanding of how wider scale and sustained impact can be achieved.

**Results:**

Overall, the resource was positively received by school staff and young people engaged well during the lesson. As a result of the stakeholder networking activities, the research team worked with the Personal, Social, Health and Economic (PSHE) Association to adapt the materials to meet their Quality Assessment and incorporate elements, such as more interactive activities, requested during the implementation study.

**Conclusion:**

The EDUCATE resource has the potential to change practice by enhancing information provision about the HPV vaccine in schools and supporting young people nationally to make informed decisions. Key learnings from the project include the importance of integrating input from target users at all stages of the research process, pragmatism in relation to evaluation research designs, and incentivising researchers to undertake translation activities through further funding and a greater focus on impact. Together, these can help facilitate the availability of public health resources and their adoption into ‘real-world’ practice.

What this study adds:

- This study reports the initial use of the EDUCATE resource to support young people's decision-making about the HPV vaccine in schools and the process involved to support wider implementation.
- The EDUCATE resource appeared to be positively received by school staff, and young people engaged well during the lesson.
- The EDUCATE resource is now available in the public domain and has the potential to change practice by enhancing information provision about the HPV vaccine in schools,.

Implications for Policy and Practice:

- By taking a more pragmatic approach to implementation evaluation the researchers were able to avoid withholding a potentially useful resource from the intended users.
- The researchers developed a public health resource considered fit for purpose and with utility for ‘real-world’ practice by integrating input from key stakeholders and target users at all stages of the research process.
- Further funding and a greater focus on impact could help incentivise researchers to undertake translation activities to ensure research innovations are made available in practice.

## 1 Introduction

### 1.1 The English HPV vaccination programme

Human papillomavirus (HPV) is a common infection spread by skin-to-skin contact which can lead to the development of cancers affecting both genders. The English schools-based HPV vaccination programme is offered to young people aged 12–13 years [1]. The Covid-19 pandemic caused disruptions to delivery and uptake has fallen to below 70 % (with substantial range in performance by local authority from 34% to 93%) [2]. Persistent lower uptake has also been identified among some populations, including minority ethnic groups and young people educated in alternative education provisions [3,4].

Schools are a widely acceptable setting for delivery of the vaccine programme [5]. However, challenges in communicating evidence-based messages exist as school immunisation teams have limited opportunities to interact face-to-face with young people, or to frame and target specific HPV vaccine messages [6,7].

### 1.2 Overview of co-production of the EDUCATE resource

The EDUCATE resource was co-produced to address young people's unmet information needs about the HPV vaccine [7]. The content, style and format were informed by the preferences of young people from disadvantaged backgrounds and diverse ethnic groups and stakeholders. The EDUCATE resource comprised a PowerPoint presentation, interspersed with five short films and a guidance document for professionals delivering the lesson (e.g. immunisation nurses, school staff) [6]. Following completion of the project, unanswered questions remained in relation to how the EDUCATE resource would be implemented in practice, who could take responsibility for delivery of the resource, and how the resource could be made publicly available.

### 1.3 Translating public health research into practice

Despite increased investment into public health research [8], evidence from research often does not translate into changes in practice [9]. Further, the length of time for public health research to change policy and practice can span decades [10]. Passive approaches to dissemination (e.g. publication in academic journals) are largely ineffective in changing practice, but often used by researchers [11]. There is increasing emphasis on implementation science to support more rapid uptake of research into ‘real-world’ practice [12], as well as developing new ways to rapidly develop effective interventions and messaging by integrating co-production methods with experimental, quasi-experimental and real-world evaluation [13].

This manuscript aims to address the limited literature describing the translation of public health research into practice. The specific objectives of the implementation and knowledge mobilisation research are to: (i) provide a case study which reports on the translation of public health research into practice; (ii) explore the feasibility and evaluate the implementation of the EDUCATE resource in schools, and; (iii) identify and engage with a wide variety of relevant stakeholders to support the sharing the of the resource.

## 2 METHODS

### 2.1 Implementation evaluation: Stakeholder meetings

Two key stakeholder meetings were held to inform the subsequent implementation activity, involving academics, representatives from school immunisation teams, NHS England, UK Health Security Agency, a local authority, and a pharmaceutical company. During the workshops, barriers and facilitators to implementing the EDUCATE resource in schools were discussed and an implementation plan developed.

### 2.2 Implementation evaluation: Setting and recruitment

Research activities were undertaken in three local authorities in the South West of England. School recruitment and data collection occurred between April–July 2022 and September–December 2022, during the 2021/22 and 2022/23 HPV vaccination programme years.

Schools with lower uptake of the HPV vaccination programme in comparison to the average within the study area in previous years were identified in collaboration with the local school immunisation team through their vaccination records and invited to participate by email. Of the 23 schools identified in April 2022, two schools consented to take part. Of the 52 schools identified September 2022, three schools agreed to participate. School recruitment began once delivery of the 2021/2022 HPV vaccination programme had already started. As a result, less schools were eligible to be invited to participate during the first round of recruitment, because the resource needed to be delivered before the vaccination session had taken place within the school. The study researcher liaised with the school contact at each participating school to discuss how the EDUCATE resource would be delivered in the school and by who.

### 2.3 Implementation evaluation: Data collection

It was anticipated that in each participating school, the delivery of the EDUCATE resource would be observed and detailed field notes relevant to implementation would be recorded. Where possible, young people who attended a lesson observed by the study researcher were invited to participate in an interview. Young people were interviewed alone, or in a small group, depending on their preferences. Key staff at each school who were involved in the delivery of the EDUCATE resource were also invited to be interviewed. Semi-structured topic guides were used to explore experiences of delivering or receiving the EDUCATE lesson.

All interviews were digitally recorded once permission had been obtained. Recordings were transcribed verbatim and transcripts anonymised. Interview participants received a gift voucher to thank them for their time.

Due to the pragmatic and time-limited nature of the evaluation, in-depth qualitative analysis was not conducted. Instead, data from observations and interviews were reviewed and summarised into descriptive themes with the aim of identifying ways in which the content and delivery of the EDUCATE lesson could be improved ahead of wider-scale rollout.

### 2.4 Knowledge mobilisation activities

We contacted stakeholders in the policy, health, education and voluntary sector with the purpose of identifying key individuals in the position to influence appropriate policy, identify possible policy levers to encourage use of the EDUCATE resource, and to enhance understanding of how sustained impact could be achieved. This involved utilising the contact networks of our local project partners to identify opportunities for engagement at a regional and national level.

The outcomes of discussions with stakeholders were captured in a matrix which summarised a list of key contacts and the opportunities for influencing policy decisions. This information was used to guide dissemination and knowledge mobilisation activities aimed at making the EDUCATE resource available more widely.

## 3 RESULTS

### 3.1 Implementation evaluation: Stakeholder meetings

During stakeholder meetings, practical barriers were identified in relation to the capacity of the school immunisation teams to deliver the lesson at scale. Further, it was suggested there was the potential to deliver the EDUCATE resource as part of the ‘Health & Prevention’ module within the statutory Personal, Social, Health, and Economic (PSHE) curriculum in schools. Through stakeholder input, it became apparent that it would likely be school staff who would be best placed to deliver the resource.

### 3.2 Implementation evaluation: Research activities

Five schools agreed to support the implementation of the EDUCATE resource. A researcher (HF) observed delivery in four schools, and obtained feedback from two school staff members and 15 young people at three schools. Overall, young people interviewed ensured the perspectives of non White British (n = 5), males (n = 5), and Year Eight students (n = 13) were included. Most of the data collection activities (interviews with one school staff member and ten young people) took place in one school.

Data collection proved to be more challenging than anticipated and only two interviews were undertaken with school staff. We had also intended to interview students after the school-based HPV vaccination session had taken place to gather information related to their involvement in consent procedures and confidence to be vaccinated. However, this was only possible in one school where the HPV vaccination session was scheduled within the timescales of the study. Therefore, we were limited in being able to collect data in relation to these topics.

The EDUCATE resource was usually provided to Year 8 students in four schools ahead of the scheduled HPV vaccination session for the academic year. In one school, the resource was delivered to Year 7 students. In four schools, the EDUCATE resource was incorporated within the PSHE curriculum and delivered by school staff with existing responsibility for that subject. In the other school, the lesson was delivered within an English lesson by the Head of Year due to close proximity of an upcoming HPV vaccination session.

Overall, the resource appeared to be well received and researcher observations confirmed that young people generally engaged well during the lesson. Young people who were interviewed were able to recall key HPV vaccine information (e.g. levels of protection offered) and valued the opportunity to learn about HPV and the HPV vaccine: ‘*It was just quite informational*’ [Young person 3, male, White British]. Two young people interviewed indicated the topic area made them feel uncomfortable: *‘I don't like the bits with the disgusting stuff [cartoon images of body images affected by HPV-related cancers], when you learn about puberty*’ [Young person 11, female, Somali].

### 3.3 Addressing young people's information needs

Observations confirmed questions were asked to the member of staff delivering the resource and the majority of young people interviewed indicated they were comfortable to do this in the classroom setting. In one of the schools, a ‘questions box’ facilitated anonymous questions as suggested within the guidance manual. A few young people interviewed commented this could have been helpful for other students: ‘*I didn't need to ask any questions but for people that maybe didn't understand it as much it could have been a bit better [if there was a confidential space to ask questions]*’ [Young person 07, female White British].

Questions covered broad related areas, often focussing on the practicalities of getting the HPV vaccine (e.g. when the vaccination session would take place, young people's consent). School staff indicated that the guidance document helped equip them to respond to young people's questions. Questions from students arose in two of the schools relating to the transmission of HPV through oral sex. The responses from school staff to address these questions differed. In one school, the school staff member answered this questions, whereas in the other school it was not felt appropriate to answer as the resource was delivered to Year 7 students: ‘*A couple of the students asked me how you get HPV in your mouth, but I kind of parked the question, and was like, ‘We'll talk about that another time,’ because I didn't want to overload the students*' [School staff member, 02].

### 3.4 Delivery of sensitive content

The EDUCATE resource features a film of an HPV-related cancer survivor sharing his experiences and was included as a direct response to suggestions from young people. After watching this film, students were given an opportunity to reflect and staff were provided with guidance to support students. Researcher observations suggested that students were often moved by the film, but did not appear to be unduly distressed by the content. However, one student interviewed acknowledged the potential to cause distress: ‘*It reminded me of … because that [experience of cancer] happened in my family, and it just reminded me of that so I guess that could be a bit personal for some people*’ [Young person, female, White British].

In one school, participation in the lesson had been discussed in advance with one student whose parent had recently completed treatment for cancer. The school staff member felt that the content of the film had been pitched appropriately as this student had not become upset during the lesson. This was despite the staff member becoming emotional themselves while discussing the film with their students because of their own parent's historic experience of cancer.

### 3.5 Adaptions to the resource

School staff made adaptions to the EDUCATE resource which included developing short activities (e.g. quizzes) ahead of the lesson to enhance engagement and learning during delivery. This was identified by one school staff members as a key area for improvement of the resource: ‘*The only thing I'd say is a lack of tasks. I think tasks are really important, otherwise it becomes a lecture, and students get disengaged by listening to my voice or anyone's voice*’ [School staff 02]. In one school, the staff member had time to test the students' learning at the end of the lesson after all the content had been covered within the allocated time. This contrasts to experiences in another school where there was insufficient time to deliver all the content, highlighting the need to ensure the resource can be used flexibly according to the needs of the students and the time available for delivery.

### 3.6 Knowledge mobilisation: Networking with local and national stakeholders

Meetings and informal discussions were held with over 80 stakeholders, including senior staff within national health organisations, international charities, education sector, local authorities, and school immunisation teams. Several routes to widespread dissemination and raising awareness of the resource were also identified (e.g. national mailing lists to healthcare professionals, relevant stakeholder groups, signposting within national guidance documents). Opportunities were also taken to promote the resource further at relevant local and regional meetings that arose through the professional networks of members of the research team.

### 3.7 Knowledge mobilisation: National implementation activities

As a result of the implementation and networking activities the research team formed a partnership with the PSHE Association who indicated an interest in hosting the resource on their website. The PSHE organisation is a national body for PSHE education and provides support to over 60,000 schools in England with resources, training, guidance and advice, and was felt by the research team to be a good way to maximise its potential reach among school staff.

The research team worked with PSHE Association to amend the resource to gain accreditation and meet the PSHE Association Quality Standard. Through this process, the EDUCATE resource was repackaged so that it would fit with learning objectives and curriculum requirements. Additional activities were incorporated to make the resource more interactive and meet the expectations of teaching staff. This also involved removing the film covering the story of an HPV-related cancer survivor from the resource as it was felt that using a ‘worst case scenario’ would take away from the key learning of the lesson. The changes to the resource recommended by the PHSE Association were overall consistent with the feedback received during the implementation evaluation.

In January 2023, the EDUCATE resource was made freely available to members of the public on the PSHE Association website [14]. The resource was promoted to the PSHE Association membership and through the stakeholder network established during the implementation phase. National guidance published by the UK Health Security Agency was updated to include sign-posting to the resource [15,16]. At the end of June 2023, the resource had been downloaded 1419 times by members of the public (including school staff). Assuming each downloaded resource is taught to one class of 30 students, the resource will have reached 42,570 students during the 2022/23 HPV vaccination programme year.

## 4 Discussion

In this paper, we provide an overview of the implementation activities which led to the refinement and wide-spread availability of the EDUCATE resource. The length of time from study inception to resource availability was four years. Although the primary focus has been for school staff to deliver, there is interest outside of the school setting and the resource could be used in a targeted way by school immunisation team to schools with sub-optimal uptake. We acquired funding to develop a bespoke website to host the EDUCATE resource, alongside materials we have developed to support parents’ decision-making about the HPV vaccine [17], to maximise usage by healthcare professionals and provide direct access to families.

Next, we reflect on the challenges of undertaking implementation research and key lessons learned by the project team through the research development and implementation process.

### 4.1 Evaluation study design

The ‘hierarchy of evidence’ of health-related research evidence places greatest weight on evidence from systematic reviews of randomised controlled trials (RCT) [18]. However, there is increasing recognition that RCTs within the field of public health can be costly, lengthy, complex and scientifically challenging to undertake [[19], [20], [21]]. Further, research evidence is the least frequently used form of information in public health policy and programme decision-making [22].

An ongoing challenge for public health researchers is determining when scientific evidence is sufficient for action. In this case, the academic team considered potential study designs to establish effectiveness of the EDUCATE resource at improving uptake of the HPV vaccination programme. This was weighed against the potential gains of enabling a ‘low-risk’ educational resource to be used in practice more rapidly. By taking a more pragmatic approach we were able to avoid withholding a potentially useful resource from the intended users.

### 4.2 Meaningful partnership working

A flexible approach to engaging with key stakeholders and young people was integrated at all phases. For example, at the study inception phase feedback was gathered by testing the initial prototype with the target population in schools. At the later stages, the researchers dedicated time to building relationships with key stakeholders to support promotion of the resource once available in the public domain. By being responsive to the input and experience of key stakeholders and young people, the researchers developed a public health resource with utility for ‘real-world’ practice.

### 4.3 Undertaking research in schools

The research team faced significant challenges to undertaking research activities within the school setting, with over 50 schools approached in a two-stage recruitment strategy. We originally planned for the EDUCATE resource to be delivered in an additional research site, but it was not possible to recruit any schools in this setting. Recruitment barriers may have been influenced by the timing of recruitment post Covid-19 pandemic which was a challenging period for schools.

There were limited windows of opportunities to timetable delivery of the EDUCATE resource (e.g. prior to vaccination session) and difficulties in engaging with school staff to organise the research activities. For example, one school delivered the EDUCATE resource without informing the researcher and no observations could take place and subsequent planned interviews were not undertaken. Understanding of research processes and capacity of staff to engage in research, given workload pressures and competing priorities, appeared much more limited in schools than is common in healthcare organisations. Addressing these issues will be crucial to unlocking the potential of schools to support delivery of evidence-based public health interventions and could be achieved by providing greater financial incentives to participate in research projects that align to the priorities of the schools.

### 4.4 Need for creating a research environment to support research translation

In recent years, grant opportunities from funding bodies have increased for implementation and knowledge mobilisation activities. The work undertaken as part of this study demonstrates that a modest amount of funding can be helpful to close the evidence-practice gap and ensure research efforts are not wasted. Activities to translate research findings into practice demand significant time from researchers, but do not usually generate additional funding or publications which current academic pathways tend to reward [23]. Additional funding opportunities, alongside a greater focus on impact as an important outcome of academic research, could further incentivise academics to make research innovations available in practice.

### 4.5 Limitations of the study

Despite best efforts, managing the research activities with schools was challenging and we were unable to recruit to target school staff and young people to participate in an interview. As a result, an in-depth approach to qualitative data analysis was not possible for this study. The researcher who led the research activities was also involved in the development of the EDUCATE resource which has the potential to introduce reporting bias to the findings, as well as participant bias. However, the conduct and the initial findings of the study were discussed with the wider project team throughout the project to mitigate this. Finally, the research activities were undertaken in one geographical area within the South West of England and therefore the findings may not be generalisable to other parts of the country.

## 5 Conclusion

The EDUCATE resource has the potential to change practice by enhancing information provision about the HPV vaccine in schools and support young people nationally to make informed decisions whether to be vaccianated or not. Key learnings include the importance of integrating input from target users at all stages of the research process, pragmatism in relation to evaluation research designs, and incentivising researchers to undertake translation activities. Together, these can help facilitate public health resources become available and adopted into ‘real-world’ practice.

## Ethics approval and consent to participate

The University of Bristol Faculty of Health Science Research Ethics Committee (reference: 10551) provided approvals for the implementation study. Informed written or verbal assent/consent was obtained from prior to participating in an interview. All interviewees aged 16 years or older gave written informed consent before participating in the study. For participants aged younger than 16 years, both parental consent and affirmative young women's assent were required prior to participation.

## Consent for publication

Not applicable.

## Data availability statement

The datasets generated and/or analysed during the current study are not publicly available but are available from the corresponding author on reasonable request.

## Authors’ contributions

HF wrote the first draft of the manuscript. HF led the implementation activities, with support from LL, CT, AT. All authors (HF, SA, TC, MD, KE, LH, MH, LL, AT, CT) contributed to the final version of the manuscript. All authors read and approved the final manuscript.

## Funding statement

This project was funded by the University of Bristol ESRC Impact Acceleration Account (2019-23) (ES/M500410/1) and PolicyBristol from the Research England QR Policy Support Fund (QR PSF) 2021-22.

## Declaration of competing interest

The authors have no conflicts of interest to declare.

## List of abbreviations

HPV: Human papillomavirus
PSHE: Personal, social, health and economic
RCT: Randomised controlled trial
